# The Role of Individuality in the Future of Aesthetic Medicine

**DOI:** 10.1111/jocd.71152

**Published:** 2026-09-14

**Authors:** Jonquille Chantrey, Evan Rieder, Carola de la Guardia

**Affiliations:** ^1^ ØNE Aesthetic Studio Cheshire UK; ^2^ 36 North Moore Dermatology, New York New York USA; ^3^ Allergan Aesthetics, an AbbVie company Madrid Spain

**Keywords:** aesthetics, sociologic factors, surveys and questionnaires

## Abstract

**Background:**

Aesthetic medicine has gained wider acceptance across a range of demographics and geographic regions. Individuality, an important driver of aesthetic procedures, is a complex concept associated with a unique combination of attributes that express a person's identity, closely linked to self‐confidence and self‐image.

**Aims:**

There is a need to understand emerging trends in aesthetics and individuality as a central theme in the future of aesthetic medicine.

**Methods:**

An extensive, multifaceted, global research project was conducted from 2021 to 2023 to gain an understanding of emerging signals and trends across aesthetics and to better understand the concept of individuality and its role in aesthetic procedures. The study employed a combination of qualitative and quantitative research methods, including desk research, social listening, interviews, and a consumer survey with 6 503 respondents from 12 countries, to explore aesthetic trends and the concept of individuality in relation to beauty and aesthetics.

**Results:**

Results indicated that individuality is a multidimensional concept and a key factor in defining beauty and highlighted the importance of personalized aesthetic medicine. Aesthetic treatments and services should reinforce patients' individuality and self‐expression through more representative and customizable product and service options. Expert interviews reinforced the need for greater inclusivity in aesthetic practice.

**Conclusions:**

Personalized aesthetic medicine with an individualized approach can have important psychosocial and emotional benefits for patients that lead to improvements in well‐being, emotional attributes, and social attributes. These findings support a shift toward treatment models that are inclusive and tailored to the unique goals and identity of each patient.

## 1 Introduction

Global statistics reveal a growing acceptance of aesthetic procedures across races, ethnicities, cultures, ages, and genders (i.e., a “normalization of aesthetics”) [1, 2, 3]. According to the International Society of Aesthetic Plastic Surgery (ISAPS), overall nonsurgical aesthetic procedures increased nearly 40% to 20.5 million worldwide, while surgical procedures grew more than 46%, with increases reflected across a broad range of demographic categories and geographic regions from 2020 to 2024 [3].

Traditionally associated with a desire to repair an anatomic defect or achieve universal ideals of beauty and youthfulness, aesthetic procedures are now often utilized to meet individual appearance expectations as well as to attempt to keep pace with aesthetic standards for gender identity, age, ethnicity, race, skin type, socioeconomic status, and other demographic characteristics [4, 5, 6, 7, 8]. Underlying all of these motivations is a fundamental concept: self‐image. Self‐image can encompass an individual's internal perception of their own physical appearance: how they believe they look to themselves and others, informed by both objective features and subjective perceptions. Self‐image may be influenced by innate beliefs, personal experiences, cultural norms, and social feedback. For the purposes of this research, individuality refers to the distinctive, multifaceted, and unique combination of physical, emotional, and social attributes through which a person expresses their identity in aesthetic contexts. It is shaped by self‐confidence, self‐esteem, and cultural context. In aesthetic medicine, self‐image reflects an innate, natural, and unique presentation specific to the individual, rather than conformity to standardized ideals.

For most individuals, the appearance of the face is a central component of self‐image [5]. Consequently, interest in facial aesthetic procedures has increased globally compared with other cosmetic procedures [9]. According to the ISAPS, facial aesthetic procedures were among the top 5 most common surgical and nonsurgical procedures performed in 2024 [3]. Approximately 7.5 million surgical facial procedures and 18.1 million nonsurgical facial procedures were performed worldwide [3]. With an ever‐growing number of facial aesthetic procedures and greater availability of facial aesthetic products, improving patients' self‐image, which can translate into improved self‐confidence, is vital to the success of facial aesthetics [10]. Feeling unsatisfied with one's facial appearance, desiring to increase self‐confidence, and wanting to achieve a younger or “less tired” appearance are key factors that influence people's decisions to undergo facial aesthetic procedures [11, 12, 13, 14]. These factors have become more important than ever with the rise of global interconnectedness, the ascent of social media and videoconferencing, and the availability of photo filtering and technology allowing the portrayal of an idealized self to the world. Discussions of individuality in aesthetics should address how these digital environments mediate self‐image and identity.

For more than 10 years, social media apps and platforms have offered features to enhance users' appearances in a variety of ways, including removing blemishes and wrinkles from the skin, widening the eyes, and changing the contour of the jaw [15]. Today, social media is a very popular forum for social interaction and self‐expression [16], and it is common to post photographs, including self‐portraits, online [14] and use filters and other augmented reality tools to influence how users view themselves and how others view them [16]. An ultimate goal, commonly, is to convey an ideal version of one's physical self [15, 17]. Such forms of self‐expression and social interaction, however, have been shown to lead users to scrutinize themselves more intensely [18] and to experience worsening self‐image, self‐confidence, and possibly psychiatric illness [14].

Individuals have also become more focused on their self‐image, particularly their facial appearance, because of the increased use of videoconferencing since the COVID‐19 pandemic [19]. Videoconferencing platforms have given people unedited views of themselves in motion, which most are not accustomed to; videoconferencing, unlike in‐person conversations, involves viewing oneself speaking and moving in real time and having one's face displayed alongside the faces of others [17]. This is further compounded by limitations of the camera, including poor resolution, lighting, and angles, which can skew the features of the face. For some, seeing their image on videoconferencing is discordant with their appearance in person, which may cause them to think that their self‐perceived facial flaws are more noticeable than they physically are, potentially negatively impacting their self‐image [20, 21].

There has also been a surge in the number of transgender patients who seek facial aesthetic procedures along with forms of gender‐affirming care so that their physical appearance aligns with their self‐identity and self‐image [22]. Further, factors such as culture and ethnic background shape perceptions of beauty and appearance and affect self‐image [5, 23, 24, 25]. With increasing numbers of individuals from diverse demographic sectors and identifiers now pursuing aesthetic procedures, some individuals may feel underrepresented by the available selection of treatments and approaches for their aesthetic care [26].

Within this milieu of evolving motivations for aesthetic treatment, patients often present with preconceived ideas of the facial aesthetic procedures they want performed and seek out providers to administer them [27]. There is also awareness of the psychological benefits that facial aesthetic treatments can have; the impact on mental health can potentially affect everyone, regardless of culture or geography [12].

Supporting patients' expressions of aesthetic individuality and synchronizing clinical practice with each unique patient's needs is now an important area of attention for aesthetic practitioners [7]. To that end, the present analysis aimed to elucidate the trending concept of individuality in relation to beauty and aesthetics, as well as bridge the gap between consumer‐perceived needs and practitioner‐delivered outcomes.

## 2 Methods

An extensive, multifaceted, global research project was undertaken beginning in 2021 to gain an understanding of emerging signals and trends across aesthetics. This initiative was led by Allergan Aesthetics, an AbbVie company, in collaboration with independent agencies specialized in trends forecasting and market research. This study was exempt from institutional review board (IRB) approval in accordance with 45 CFR 46.104(d) [2].

**FIGURE 1:**
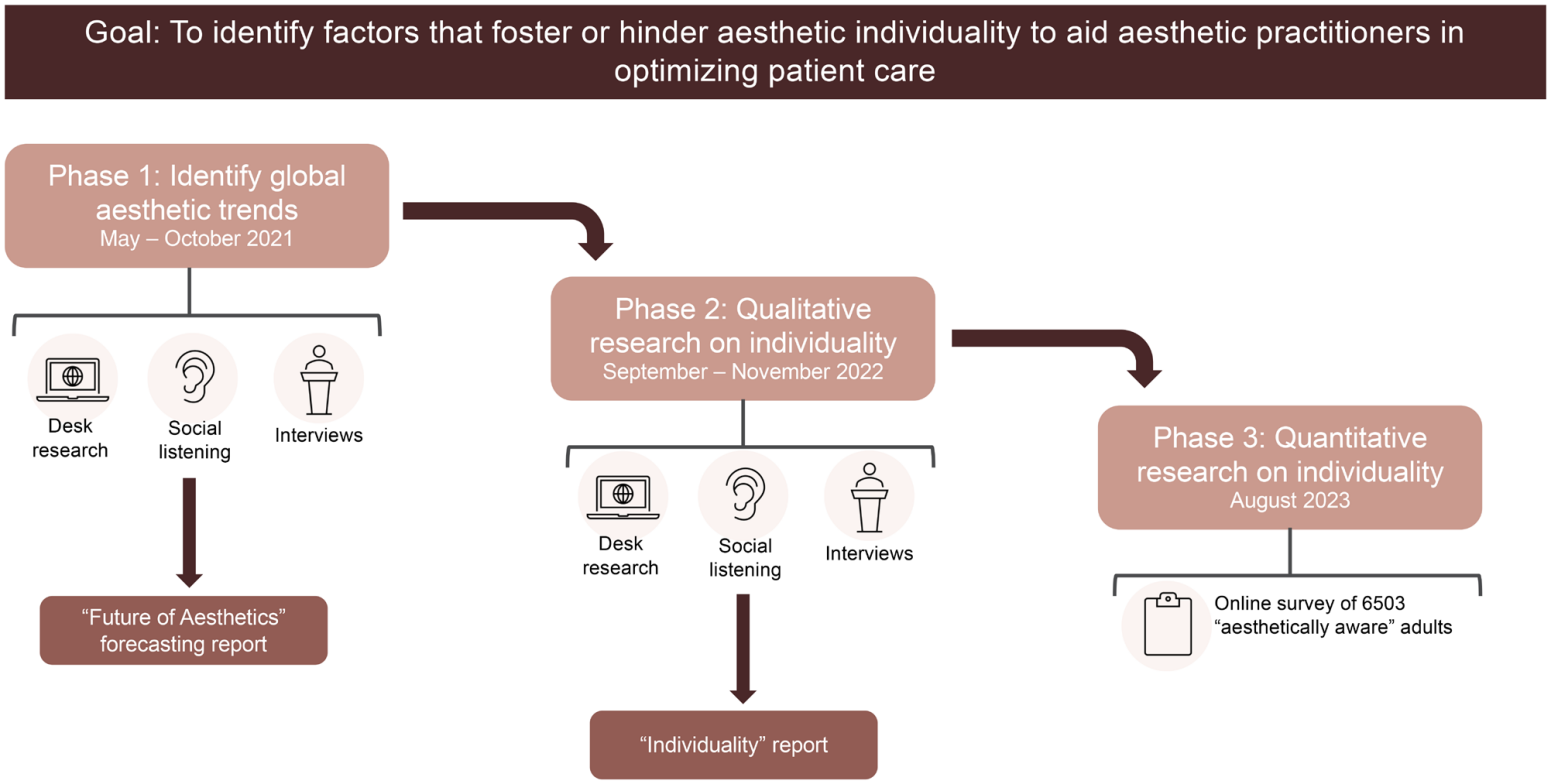
Study design.

This staged research project consisted of international field and desk research with both qualitative and quantitative methodologies (Figure 1). Desk research included internet research conducted across consumer and business media, as well as market and industry reports in multiple geographies, specifically Australia, Brazil, China, India, Japan, Russia, South Korea, the United Arab Emirates, the United Kingdom, and the United States.

The first phase of research, conducted from May to October 2021, aimed to identify global aesthetic trends. This research utilized qualitative methods such as desk research (which included reviewing existing marketing data), social listening (research conducted on specific major social media platforms, blogs, forums, and online news sources), and interviews. This phase of research culminated in the identification of 10 aesthetic consumer trends and 4 trend drivers (the underlying forces behind the trends). These informed the development of a “Future of Aesthetics” forecasting report. “Diverse individuality” was found to be a common intrinsic theme of the trends identified.

Qualitative supplementary collaborative research, focused on the theme of individuality, was conducted between September and November 2022; this phase included desk research, social listening, and interviews. Extensive social listening research was conducted on mentions of “individuality” in specific major social media platforms, blogs, forums, and online news sources. Four leading practitioners and experts in the aesthetics industry from Brazil, Japan, the United Kingdom, and the United States, as well as 4 experts in other research fields (psychology, beauty, marketing, and futures) from Australia, Brazil, and the United Kingdom, were interviewed on the topic of aesthetic individuality and the factors that impact and influence it. This phase of research produced an “Individuality” report.

Quantitative research was then conducted in August 2023 to define the concept of individuality and its components, identify specific individuality themes related to aesthetics, and understand the brands and consumer forces behind individuality. To collect this information, a 20‐min online survey was created using a data collection portal, IBM SPSS Dimensions. Extensive pre‐field quality checks were conducted to ensure consistency, accurate data capture, and routing. Once the IRB exemption was obtained and the quality check was complete, a soft launch of the survey was conducted on 10% of the study population to ensure accuracy before release to the remaining study population. As part of this research, 6 503 adults from 12 countries (Australia, Brazil, Canada, China, France, Germany, Italy, Japan, Saudi Arabia, Spain, the United Kingdom, and the United States), aged 25 to 65 years with a 2:1 ratio of female to male and screened as being “aesthetically aware” based on preliminary questions, provided their informed consent and then responded to the survey. Participants identified as “aesthetically aware” agreed with 2 of 3 of the following statements: “It is important to me to look good for my age,” “I care about improving the appearance of my face and body,” and “Spending money on improving the appearance of my face and body is worthwhile”; were aware of at least 1 beauty treatment; and would consider having a beauty treatment in the future. Survey questions were based on a 5‐point scale (1 = strongly disagree, 2 = somewhat disagree, 3 = neither agree nor disagree, 4 = somewhat agree, 5 = strongly agree), where those responding with a 4 or 5 were considered in agreement with the statement. Overall total results were weighted based on a country average approach with an equal weighting across all markets included to control for differences between sample sizes. This approach was selected to ensure global insights were not disproportionately driven by the larger US sample. Significance testing was conducted at a 95% confidence level. This research led to the development of additional market‐oriented materials.

Data gleaned from all of these research approaches were distilled, analyzed, interpreted, and summarized by an independent agency to identify the internal and external factors that foster or hinder aesthetic individuality, whether cultural, biologic, technologic, or social, to aid aesthetic practitioners in optimizing patient care.

## 3 Results

**FIGURE 2:**
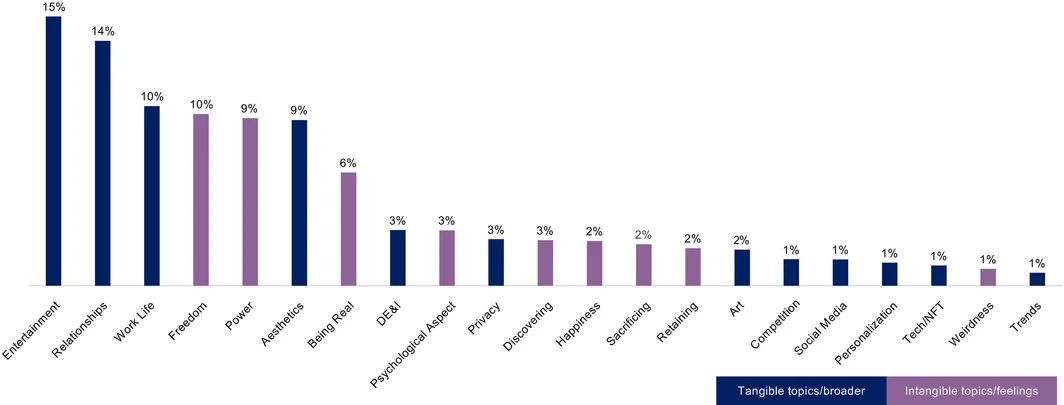
Overview of social listening conversation themes related to individuality (*N* = 637 168). DE&I, diversity, equity, and inclusion; NFT, non‐fungible token.

**FIGURE 3:**
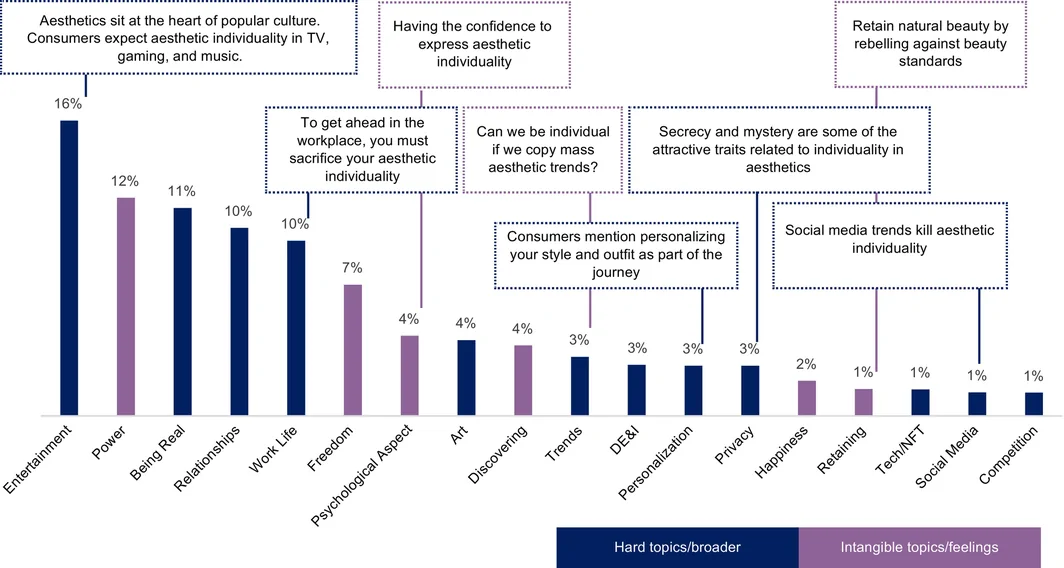
Social listening conversation themes and subtopics related to aesthetic individuality (*N* = 37 552). DE&I, diversity, equity, and inclusion; NFT, non‐fungible token.

Social listening conversations related to individuality revealed that there is no single definition of individuality; it is a versatile concept encompassing positive attributes that often find expression in the areas of entertainment, relationships, and work life (Figure 2). Social listening conversations specifically related to aesthetic individuality demonstrated that aesthetic individuality is a complex issue pertaining to discussions of style, fashion, hair, and associated topics. Aesthetic individuality conversations reflected the same themes as general individuality conversations (Figure 2), with several major underlying subtopics, which included the roles of popular culture, the workplace, natural beauty, and self‐confidence (Figure 3).

**FIGURE 4:**
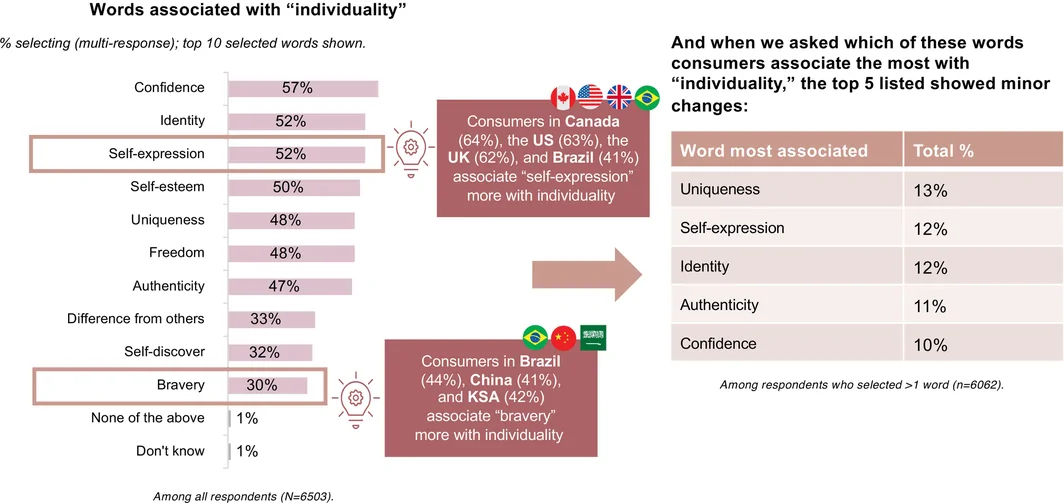
Consumer survey results for terms associated with individuality. KSA, Kingdom of Saudi Arabia.

Consumer survey responses confirmed the complexity of defining individuality based on the words respondents associated with individuality, with confidence, identity, self‐expression, and self‐esteem scoring high on the list of defining terms (Figure 4).

**FIGURE 5:**
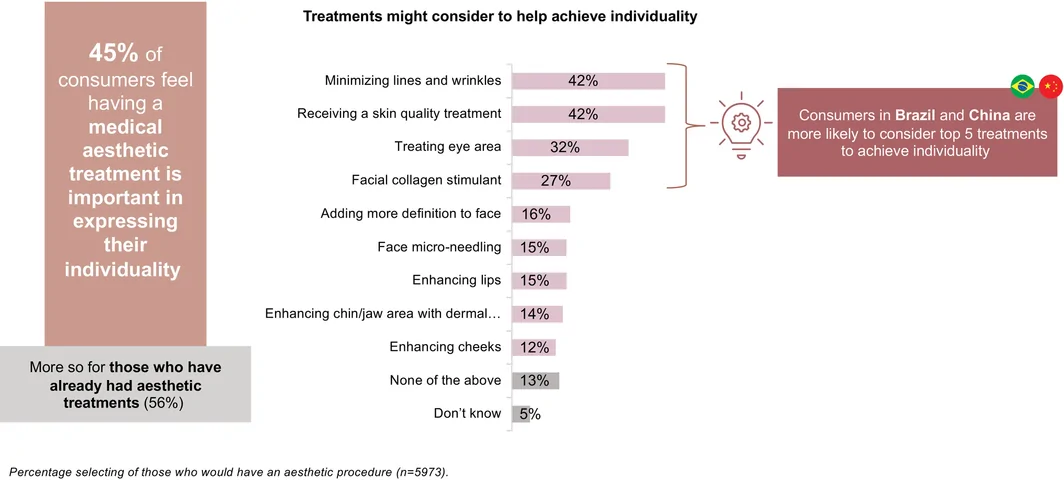
Consumer survey results for achieving individuality through aesthetics.

**FIGURE 6:**
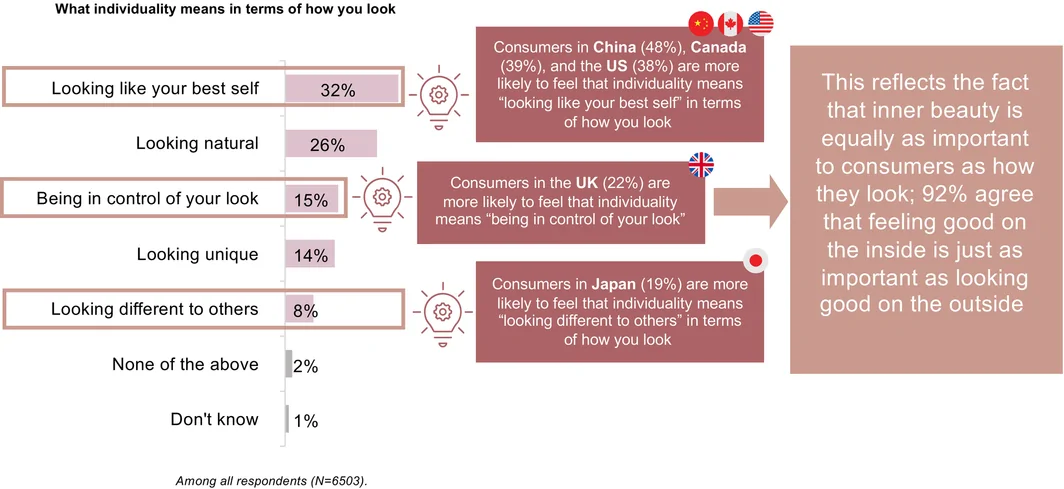
Consumer survey results for the meaning of individuality to appearance based on 6 503 respondents.

**FIGURE 7:**
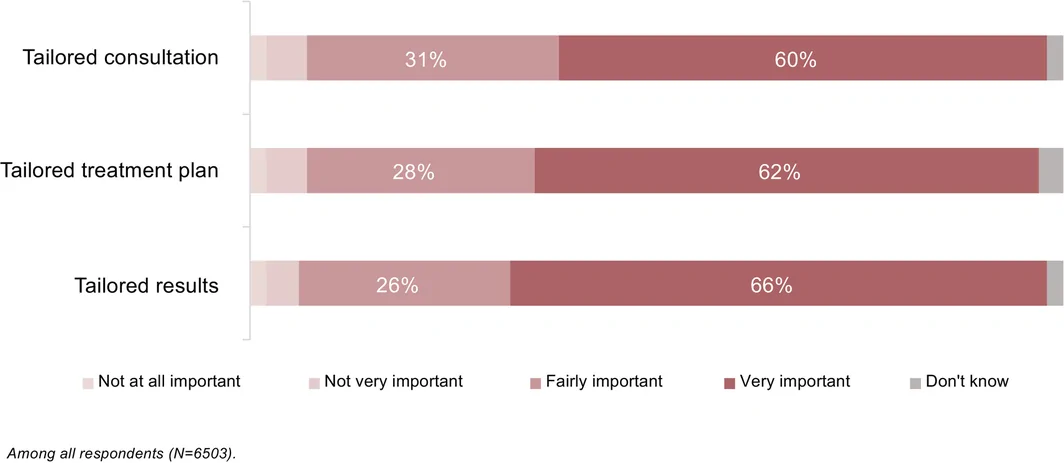
Consumer survey results for the importance of personalized aesthetic treatment.

When asked directly, 68% (95% CI = 66.9% to 69.1%) of the 6 503 consumer survey respondents agreed that individuality stands as the paramount factor in defining beauty. As such, to achieve individuality, 45% (95% CI = 43.8% to 46.2%) of respondents indicated that receiving medical aesthetic treatment was important, with priority given to treatments for fine lines, skin quality, and the periorbital region (Figure 5). Consumer survey responses also indicated that an appearance that reflects individuality is one that communicates a person's “best self” and a natural look (Figure 6). At the same time, consumer survey responses suggested that an individual's best self is unique to that individual, and personalized consultations, treatments, and outcomes remain critical in the context of medical aesthetics (Figure 7). A key finding suggested by this research, therefore, was a need for personalized aesthetic medicine with an individualized approach. This suggests that self‐expressive treatments may yield higher patient satisfaction than those aimed at conforming to standardized ideals.

Interviews with aesthetic experts and practitioners revealed opinions and concepts that aligned with those expressed by social listening and consumer research. A common theme of these interviews, e.g., was the need for treatment approaches tailored to each individual (i.e., personalized aesthetic medicine). The interviewees emphasized the importance of treating the unique face of every patient, regardless of background or heritage, in an individualized way. These respondents also affirmed that inclusion is a growing trend in aesthetic medicine, and 68% (90% CI = 66.5% to 69.5%) of the 2 500 respondents reported that they believed that current marketing and advertising standards of beauty are too rigid with regard to diversity and inclusion.

The aesthetic experts and practitioners also underscored that the patient consultation is the pivotal moment in which to affirm that the patient is being seen and heard as an individual and that aesthetic treatment will be tailored accordingly, and every touchpoint thereafter provides an opportunity to give ongoing support to the individual's need for self‐expression and for the physical, emotional, and social benefits.

**FIGURE 8:**
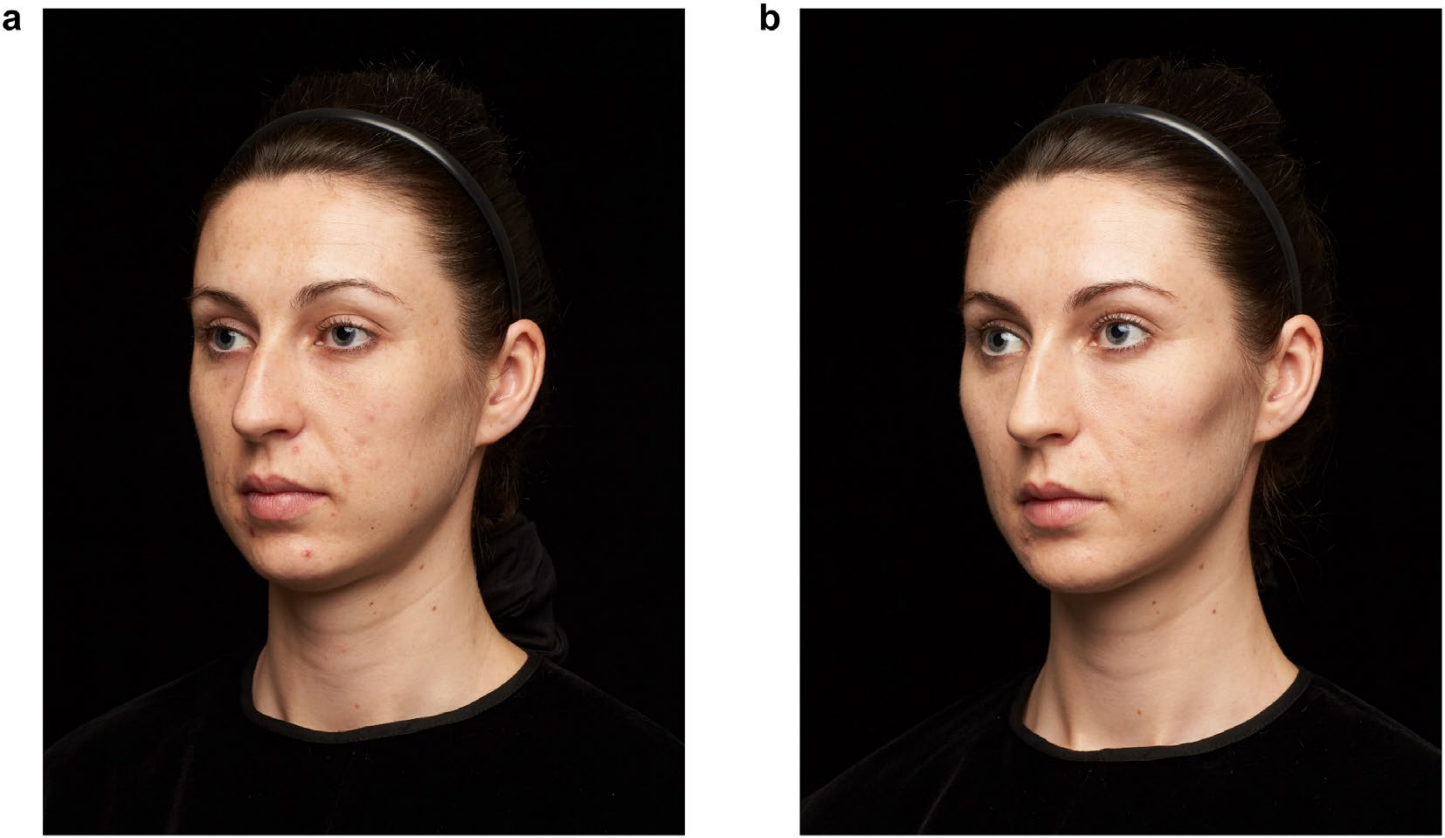
Images show a 35‐year‐old White female before (a) and 1 month after (b) treatment with 2.0 mL VYC‐12L to the forehead, cheeks, and neck; 1.0 mL to the lateral zygoma; and 1.0 mL to the gonial angle. The patient desired to minimize signs of facial aging while achieving natural results (positive aging archetype) and eradicate the signs of grief and sadness. Noted improvements were seen in skin quality.

**FIGURE 9:**
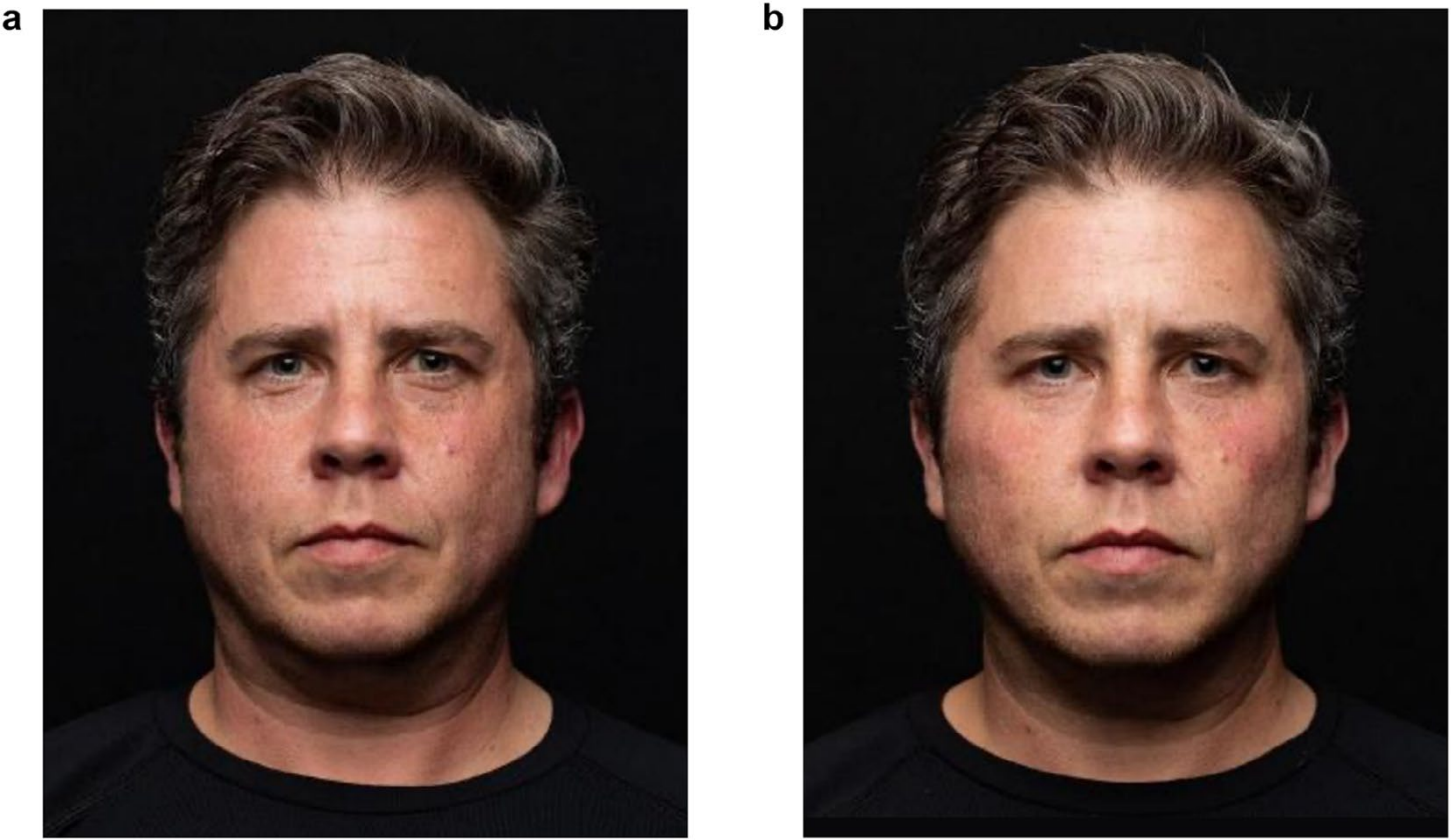
Images show a 48‐year‐old White transgender male before (a) and immediately after (b) treatment with 3.55 mL VYC‐20L, 0.8 mL VYC‐15L, and 0.65 mL VYC‐12L to the upper face. The patient desired to look and feel more masculine (transformation archetype), but also less tired. Improvements were noted in masculinity signaling, eyelid skin laxity, and canthal tone.

Representative examples of outcomes of personalized aesthetic medicine with an individualized approach are illustrated in Figures 8 and 9. Case 1 was a 35‐year‐old White female who desired to minimize signs of facial aging while achieving natural results (positive aging archetype) and eradicate the signs of grief and sadness. The patient received a total of 2.0 mL VYC‐12L to the forehead, cheeks, and neck; 1.0 mL to the lateral zygoma; and 1.0 mL to the gonial angle. Following treatment, enhancements in skin quality were noted, with reductions in acne and acne scarring, eye bags, and tear troughs; brighter, smoother, and more radiant‐looking skin; and improvements in canthal tone and early submental sagging, and less heavy nasolabial folds. Case 2 was a 48‐year‐old White transgender male who desired to look and feel more masculine (transformation archetype), but also less tired. The patient received 3.55 mL VYC‐20L, 0.8 mL VYC‐15L, and 0.65 mL VYC‐12L to the upper face. Following treatment, the face appeared stronger and more masculine, and improvements in eyelid skin laxity and canthal tone were noted.

## 4 Discussion

Aesthetics is a unique medical specialty influenced by trends that continually evolve with popular culture, public opinion, media, and politics [4, 5, 28, 29, 30]. As more people from diverse backgrounds access aesthetic medicine, understanding and representing their aesthetic objectives becomes increasingly important for practitioners, who may have preconceived aesthetic preferences that no longer reflect the array of individual patients' aesthetic concerns and goals [7]. The surveyed population included only those considered aesthetically conscious to examine the relationship between individuality and aesthetic self‐expression among participants who actively engage with aesthetics. Individuals who are more aware of and involved in aesthetic practices are more likely to provide relevant, informed, and meaningful responses regarding how individuality is expressed through appearance, style, and visual identity. Limiting the sample in this way also helped reduce irrelevant responses and supported a clearer analysis of the research topic. It is the opinion of the authors, and has been affirmed by this research, that aesthetic practitioners must be cognizant that aesthetic individuality can be expressed in many ways, and there is no one‐size‐fits‐all approach to aesthetic treatment. Aesthetic practitioners can help support individuality through their initial consultation with patients and by continually considering ways to make product and service options more inclusive, representative, and customizable to each patient. In clinical practice, this underscores the importance of developing consultation frameworks that explicitly elicit patient narratives about identity, rather than focusing solely on desired physical changes.

Large‐scale market research conducted in 17 different countries from 2014 to 2017 demonstrated that patients seeking aesthetic procedures may be categorized into 4 archetypes based on their treatment goals, motives, and requests. These archetypes include beautification (a focus on beauty, influenced by trends and social media, with a desire to enhance specific features to achieve a particular look), transformation (an emphasis on a specific aesthetic ideal to improve social status or competitive edge, often due to cultural pressure), correction (attention to improving a specific feature negatively impacting quality of life, and rebalancing or reproportioning features for increased comfort and confidence), and positive aging (concentrating on minimizing signs of facial aging while achieving natural, subtle results to portray a better version of oneself) [4].

Positive aging continues to be an important goal for individuals, as the consumer survey responses in our study have suggested, with the emphasis on giving treatment priority to fine lines, skin quality, and the periorbital region. Likewise, among aesthetically conscious people in a global physician and patient perception survey, the 3 most frequently reported cosmetic treatments or procedures were facials, professional‐grade skincare products, and dermaplaning [31]. Of 25 facial features considered, respondents were most concerned about under‐eye bags and dark circles as well as crow's feet lines and forehead lines [31].

Self‐image is an important focus for individuals. Facial appearance is a central component of self‐image [5]. A positive self‐image increases self‐esteem and overall mental health [32] and improves relationship satisfaction [33]. Cosmetic treatment creates the potential to align an individual's outer appearance with their inner self‐image, resulting in increased satisfaction with their facial appearance and improved psychological well‐being [34]. In one study, 69% of respondents stated they use cosmetic treatments to create a look that reflects how they feel inside, and 72% felt that cosmetic treatments impacted how they viewed themselves [35]. In another study, lower feelings of authenticity (i.e., the ability to present as one's real self) were associated with interest in cosmetic treatment, and patients reported increased authenticity following a minimally invasive cosmetic procedure [36]. Cosmetic procedures may improve the degree to which patients feel like their real selves; however, a small number of patients seeking cosmetic procedures suffer from body dysmorphic disorder or perception drift, where self‐image is distorted and perceived flaws in appearance are amplified, causing patients significant distress and impairment in social, occupational, and other areas of functioning [37]. These issues are further compounded by social media and the ease at which photos are manipulated, creating unrealistic beauty standards and leading to excessive use of cosmetic procedures [38].

We encourage the aesthetic practitioner to conduct a thorough consultation to take note of and affirm the patient's specific treatment goals, motives, and requests; consider their archetype(s); and aim to fulfill the patient's desire for self‐expression, confidence, and well‐being. Incorporating a structured “individuality mapping” tool into initial consultations can guide practitioners in aligning treatment plans with both aesthetic and psychosocial goals. A retrospective serial case study of a customized facial aesthetic treatment approach using hyaluronic acid fillers to treat emotional and social attributes found that, after treatment, patients reported experiencing enhancements to their overall well‐being; positive changes in their emotional attributes, including feeling less sad; and improvements in their social attributes, such as looking friendlier, more energetic, and more optimistic [39]. These positive results suggest that facial aesthetic treatments that consider individual patient characteristics can lead to overall improved well‐being.

This study was subject to several limitations. First, this survey was limited to individuals with aesthetic awareness and a desire to receive beauty treatment, which may have limited the generalizability of these findings and may have introduced selection bias, as respondents had an overly favorable view of aesthetic treatments. Additionally, there is a lack of information from the African continent, as well as limited information from the European Union following Brexit. Second, the use of online survey methodology may have further introduced selection bias, as older individuals and those with lower socioeconomic status may have been less likely to respond. Third, the cross‐sectional nature of the survey limited the ability to analyze behavior over time and represented the opinions of participants at a single time point.

Ethically, practitioners must remain vigilant to avoid reinforcing exclusionary beauty norms, ensuring that treatment recommendations support authentic self‐expression rather than societal pressure. Provided that the individual's needs are represented and reflected in the aesthetic treatment plan and approach, with the unique preferences, psychology, skin type, microbiome, phenotype, and genotype of the individual adequately acknowledged and accounted for, achieving aesthetic individuality can have important psychosocial and emotional benefits for patients.

## 5 Conclusions

Individuality is a paramount factor in defining beauty and a key driver of aesthetic procedures. It is a complex concept associated with self‐confidence, identity, self‐image, and self‐expression. An individual's best self is unique to that individual, and personalized consultations, treatments, and outcomes remain critical in the context of medical aesthetics. Future research should explore how emerging technologies, such as skin genomics and adaptive injectable formulations, may further enable individuality‐driven aesthetic medicine.

A key finding of this research was a need for personalized aesthetic medicine with a comprehensive and considered individualized approach. Medical aesthetic treatments that reinforce patients' individuality and self‐expression, and patient consultations that affirm that the patient is being seen and heard as an individual and their treatment is being tailored accordingly, provide a means to support the individual's need for self‐expression and reinforce their self‐image in a positive way. The benefits can extend beyond improvements to physical appearance to gains in emotional, social, and psychological well‐being.

These insights hold relevance not only for aesthetic practitioners, but also for allied fields such as psychology, sociology, and design thinking, where individuality and visual self‐representation are central concerns. Ultimately, prioritizing individuality in aesthetic medicine demands a commitment to seeing each patient as a partner in co‐creating outcomes that reflect their unique identity, motivations, and lived experience.

## Author Contributions

Study design: N/A. Principal investigator: N/A. Study investigator: N/A. Enrolled patients: N/A. Collection and assembly of data: N/A. Data analysis: N/A. Data interpretation: All authors. Manuscript preparation: All authors. Manuscript review and revisions: All authors. Final approval of manuscript: All authors.

## Funding

This work was supported by Allergan Aesthetics, an AbbVie company.

## Disclosure

Allergan Aesthetics, an AbbVie company, funded this study and participated in the study design, research, analysis, data collection, interpretation of data, review, and approval of the publication. All authors had access to relevant data and participated in the drafting, review, and approval of this publication. No honoraria or payments were made for authorship. Medical writing support was provided by Regina Kelly, MA, and Andrea Waksmunski, PhD, of Peloton Advantage, L.L.C. (an OPEN Health company) and funded by Allergan Aesthetics, an AbbVie company.

## Consent

All patients provided written consent for their photographs to be published.

## Conflicts of Interest

Jonquille Chantrey is the founder and CEO of ØNE Aesthetic Studio and is a consultant for AbbVie. Evan Rieder is the founder of 36 N Moore Dermatology and is a consultant for AbbVie and Merz. Carola de la Guardia is a full‐time employee of AbbVie and may own AbbVie stock.

## Data Availability

Data Sharing: AbbVie is committed to responsible data sharing regarding the clinical trials we sponsor. This includes access to anonymized, individual, and trial‐level data (analysis data sets), as well as other information (eg, protocols, clinical study reports, synopses, or statistical analysis plans), as long as the trials are not part of an ongoing or planned regulatory submission. These clinical trial data can be requested by any qualified researchers who engage in rigorous, independent, scientific research, and will be provided following review and approval of a research proposal, Statistical Analysis Plan (SAP), and execution of a Data Use Agreement (DUA). Data requests can be submitted at any time after approval in the US and Europe and after acceptance of this manuscript for publication. The data will be accessible for 12 months, with possible extensions considered. For more information on the process or to submit a request, visit the following link: https://vivli.org/ourmember/abbvie/ then select “Home”.
